# Midkine Overexpression Promotes Functional Recovery After Spinal Cord Injury by Enhancing Microglial Efferocytosis Via LRP‐1

**DOI:** 10.1002/cns.70841

**Published:** 2026-03-19

**Authors:** Yu Wang, Lu Fang, Chenyuan Zhai, Jili Cai, Xiangzhe Li, Lijuan Zong, Yao Geng, Chenchen Zhu, Cheng Sun, Manyu Dong, Yilun Qian, Yan Liu, Ying Huang, Zun Wang, Tong Wang, Wen‐Tao Liu, Qi Wu

**Affiliations:** ^1^ Rehabilitation Medicine Center The First Affiliated Hospital With Nanjing Medical University Nanjing China; ^2^ Department of Rehabilitation Suzhou Hospital, Nanjing Medical University Suzhou China; ^3^ Rehabilitation Medicine Center Suzhou Hospital, Affiliated Hospital of Medical School, Nanjing University Suzhou Jiangsu China; ^4^ Department of Rehabilitation Medicine Zhongda Hospital, Southeast University Nanjing Jiangsu China; ^5^ The Second Clinical Medical School of Nanjing Medical University Nanjing China; ^6^ Department of Rehabilitation Hengyang Medical School, The First Affiliated Hospital, University of South China Hengyang Hunan China; ^7^ Rehabilitation Medicine Department School of Acupuncture and Tuina, School of Health and Rehabilitation, Nanjing University of Chinese Medicine Nanjing China; ^8^ Jiangsu Key Laboratory of Neurodegeneration, Department of Pharmacology Nanjing Medical University Nanjing Jiangsu China

**Keywords:** LRP‐1, microglial efferocytosis, midkine, neuroinflammation, spinal cord injury

## Abstract

**Objective:**

Traumatic spinal cord injury (SCI) induces neuronal apoptosis and neuroinflammation, which exacerbate secondary damage and hinder functional recovery. Efficient clearance of apoptotic cells and modulation of the inflammatory microenvironment of spinal cord are essential for promoting tissue repair. This study aimed to investigate whether Midkine (MDK), a heparin‐binding growth factor, facilitates functional recovery after SCI and explores the underlying mechanisms.

**Methods:**

A rat model of moderate SCI was established using Allen's impact method. Lentiviral vectors were used to overexpress MDK in the spinal cord. Behavioral assessments, including BBB score and gait analysis, were performed to evaluate motor function recovery. Motor evoked potentials (MEPs) serve as a neurophysiological tool for evaluating the functional integrity of the corticospinal tract. In vivo and in vitro experiments were conducted to assess microglial efferocytosis and elucidate the underlying molecular mechanisms.

**Results:**

Transcriptomic bioinformatic analysis suggests that SCI is characterized by pronounced accumulation of apoptotic cells and robust neuroinflammatory responses, whereas single‐cell analysis implicates MDK as a key contributor to neurorepair after SCI. MDK expression is dynamically regulated following SCI, with an early upregulation followed by a gradual decline over time, its location predominantly observed around microglial cells. Functionally, MDK overexpression significantly enhances motor recovery after SCI, accompanied by reduced neuroinflammation, decreased neuronal apoptosis, and improved neuroprotection. Mechanistically, MDK promotes microglial efferocytosis both in vivo and in vitro, activates the AKT/mTOR signaling pathway, upregulates BDNF and LRP‐1 expression, and facilitates microglial polarization toward an anti‐inflammatory M2 phenotype. Notably, inhibition of LRP‐1 with receptor‐associated protein (RAP) abolished the efferocytic and neuroprotective effects of recombinant MDK, highlighting LRP‐1 as a key mediator of MDK's actions in microglia.

**Conclusion:**

Our study unveils the MDK/LRP‐1/efferocytosis axis as a previously unrecognized therapeutic target for SCI. By orchestrating apoptotic cell clearance, dampening neuroinflammation, and fostering neuroprotection, this axis critically shapes the post‐injury microenvironment to facilitate recovery. These findings suggest that MDK‐centered therapy may represent a strategy for spinal cord repair, with LRP‐1 modulation offering precise control over microglial responses.

## Graphical abstract

Midkine (MDK) promotes functional recovery after spinal cord injury by enhancing microglial efferocytosis, reducing neuroinflammation, and decreasing neuronal apoptosis. Overexpression of MDK activates the AKT/mTOR pathway, upregulates LRP‐1 and BDNF, and drives microglia toward an anti‐inflammatory phenotype. Meanwhile, blocking LRP‐1 abolishes MDK‐mediated neuroprotection, highlighting the MDK/LRP‐1 axis as a promising therapeutic target.

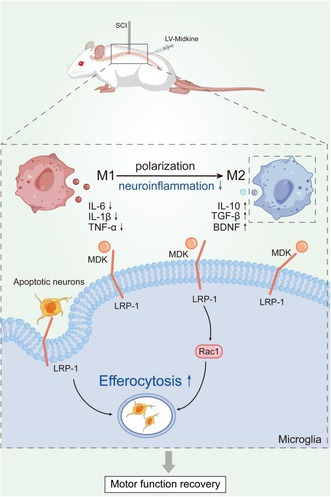

**Abbreviations**
AKT: Protein kinase B
Bax: BCL2 Associated X
BBB: Basso Beattie, and Bresnahan
Bcl‐2: B‐cell lymphoma‐2
BDNF: Brain Derived Neurotrophic Factor
CNS: Central nervous system
GEO: Gene Expression Omnibus
GO: Gene Ontology
GSEA: Gene Set Enrichment Analysis
IL‐10: Interleukin‐10
IL‐1β: Interleukin‐1β
IL‐6: Interleukin‐6
KEGG: Kyoto Encyclopedia of Genes and Genomes
LRP1: LDL‐receptor related protein 1
MDK: Midkine
MEP: Motor evoked potential
mTOR: Mammalian target of rapamycin
NEGF2: Neurite growth‐promoting factor 2
p‐AKT: Phospho‐protein kinase B
p‐mTOR: Phospho‐mammalian target of rapamycin
PTN: Pleiotrophin
Rac1: Ras‐related C3 botulinum toxin substrate 1
RAP: Receptor‐associated protein
ROS: Reactive oxygen species
SCI: Spinal cord injury
TGF‐β: Transforming growth factor‐β
TNF‐α: Tumor necrosis factor‐alpha
UMAP: Uniform manifold approximation and projection

## 1 Introduction

Traumatic spinal cord injury (SCI) is a devastating condition of the central nervous system (CNS) that often results in permanent motor and sensory dysfunction, for which effective therapeutic strategies remain limited [1]. Following the primary mechanical insult, secondary injury processes after SCI, particularly neuronal apoptosis, neuroinflammation, and edema, play a critical role in exacerbating tissue damage and impeding functional recovery [2]. These pathological cascades amplify the lesion area and worsen neurological outcomes by disrupting neuronal integrity and the surrounding microenvironment [3]. Despite advances in supportive care and rehabilitation, current interventions offer only partial symptomatic relief, underscoring the urgent need for novel therapeutic targets targeting multiple injury mechanisms.

Microglia, the resident immune cells in the spinal cord parenchyma, are essential for maintaining central nervous system (CNS) homeostasis under physiological conditions [4, 5, 6]. Following spinal cord injury (SCI), microglia undergo activation and adopt distinct polarization states: the M1 phenotype is characterized by the release of pro‐inflammatory cytokines, reactive oxygen species (ROS), and cytotoxic mediators, which exacerbate tissue damage and neuronal apoptosis; conversely, the M2 phenotype is associated with anti‐inflammatory responses, metabolic homeostasis, and neurorepair [7]. Efferocytosis, mainly involving the phagocytosis of apoptotic cells by M2‐type microglia, represents a crucial mechanism through which microglia mitigate inflammation and facilitate neural repair [8]; however, its specific contributions to functional recovery following spinal cord injury (SCI) remain poorly characterized. Our previous study suggests that microglial phagocytic capacity is impaired after SCI, leading to the further deterioration of the spinal microenvironment [9]. This dysfunction compromises tissue repair and hinders functional recovery [4]. Therefore, therapeutic strategies that simultaneously restore microglial efferocytic capacity while modulating their inflammatory polarization state may offer a promising approach to reconstruct the spinal cord's microenvironment following injury.

Midkine (MDK) [10], also known as neurite growth‐promoting factor 2 (NEGF2), is a 16‐kDa heparin‐binding growth factor that is rapidly upregulated in response to tissue injury and contribute to tissue repair [11]. MDK has been shown to exert neuroprotective effects in various contexts. For example, it can activate AKT signaling, thereby protecting embryonic stem cells from hypoxic injury [12]. In SCI models, in vivo administration of recombinant human MDK (rhMDK) has been reported to promote locomotor recovery in rats [12]. Despite these findings, the precise mechanisms by which MDK modulates the spinal cord microenvironment after SCI remain poorly understood. Our previous research revealed that impaired LRP‐1 function after SCI contributes to defective microglial phagocytosis, accumulating myelin debris, and exacerbating tissue damage. Enhancing LRP‐1–mediated phagocytosis promotes debris clearance by microglia and exerts neuroprotective effects [9]. Given that MDK is a known ligand for LRP‐1, we hypothesize that MDK may regulate microglial efferocytosis via the LRP‐1 pathway to facilitate SCI repair—a mechanism that has not yet been elucidated.

In this study, we hypothesized that MDK promotes functional recovery after SCI by enhancing microglial efferocytosis via LRP‐1, thereby mitigating neuroinflammation. To test this, we combined transcriptomic and single‐cell analyses to identify MDK as a repair‐associated factor. We performed in vivo (rat SCI model) and in vitro (BV2 microglia, primary neurons) experiments for functional and mechanistic validation of MDK. We focused on the LRP‐1/AKT/mTOR axis, microglial polarization, and microglial efferocytosis. Our findings reveal that MDK enhances microglial efferocytosis and M2 polarization through LRP‐1 to reduce inflammation and improve neurological recovery. Our study highlights the MDK–LRP‐1–efferocytosis pathway as a novel therapeutic target for SCI.

## 2 Materials and Methods

### 2.1 Preprocessing of Microarray Datasets

The microarray dataset GSE93561 was obtained from the GEO database. Three samples from the sham group and three from the SCI group (GSM2454718 to GSM2454723) were selected for further analysis. The data were preprocessed in R, where probe IDs were mapped to gene symbols based on the corresponding microarray platform annotation. For genes targeted by multiple probes, the average expression value was calculated. Differential expression analysis was performed using the limma package (version 3.60.2), and the results were visualized using ggplot2.

### 2.2 Single‐Cell RNA‐Sequencing Data Analysis

The single‐cell RNA sequencing data of SCI were derived from Gene Expression Omnibus (GEO, namely GSE172167, GSE213240). The datasets from the control and SCI groups were integrated and analyzed using the Seurat R software package (version 4.1.1). The data set was normalized, and the genes with the greatest changes were identified respectively, using the default parameters of the “NormalizeData” and “FindVariableFeature” functions. Then, principal component analysis was performed using these highly variable genes, and the significant top 20 principal components were selected to perform uniform manifold approximation and projection (UMAP) dimensionality reduction. Harmony (version 0.1.0, https://github.com/immunogenomics/harmony) was chosen to remove batch effects. UMAP was used to visualize single cells. Unbiased clustering generated eleven main clusters annotated to eleven known cell types according to canonical marker genes. Differentially expressed genes (DEGs; adjusted *P*‐value < 0.05, |log2 fold change|> 0.5) were identified by the “FindMarkers” function in Seurat. Cell–cell interactions were inferred using CellChat (version 1.1.3, https://www.cellchat.org/).

### 2.3 Enrichment Analysis

To investigate biological states and functional differences between groups and cell subtypes in SCI, we performed pathway enrichment analyses using Gene Ontology (GO), Kyoto Encyclopedia of Genes and Genomes (KEGG), and Gene Set Enrichment Analysis (GSEA) based on scRNA‐seq and microarray data. These analyses were conducted with the clusterProfiler package (version 4.12.0), using pathways and gene sets mainly from the MsigDB database (version 7.5.1). An adjusted *p*‐value of < 0.05 from logistic regression was used to determine the statistical significance of the GSEA results.

### 2.4 Animals and Surgery

All adult female Sprague–Dawley rats (weighing 200–230 g, aged 9–11 weeks) were purchased from the Animal Core Facility of Nanjing Medical University, Nanjing, China (Animal license: SYXK (Su) 2020–0022). All rats were acclimated to three per cage under controlled conditions (temperature 22°C ± 2°C, and a 12/12‐h light/dark period, 55% ± 5% relative humidity) 3 days before the experiment began. All animal experiments were approved by the Nanjing Medical University Animal Care and Use Committee (No IACUC‐1903031 and No IACUC‐2005001) and were aimed at minimizing suffering and the number of animals used.

In this study, two batches of rats were used. The first batch consisted of 35 rats, which were divided into two groups: a sham group (*n* = 4) and a spinal cord injury (SCI) group (*n* = 31). The spinal cord injury model was established using Allen's model [13]. All surgical procedures were performed under anesthesia induced by an intraperitoneal injection of 1% (w/v) sodium pentobarbital (50 mg/kg). After hair removal, a laminectomy was performed to expose the spinal cord at the T9–T10 level. A moderate, incomplete SCI model was then induced using a spinal cord impactor (RWD, China) with a force of 250 kdyn [14]. Rats in the sham group underwent laminectomy only, without contusion injury. Following surgery, all rats received an intramuscular injection of potassium benzylpenicillin (32,000 U/100 g) and had their bladders manually expressed twice daily until spontaneous urination was restored. Spinal cord tissues from segments T9–T11 (within 0.5 cm centered on the injury epicenter) were collected on days 1, 3, 7, 14, 21, and 28 post‐SCI for western blot analysis (*n* = 4 per group per time point). Additionally, spinal cord tissues from the same region were collected on day 28 for immunofluorescence staining (*n* = 3 per group).

The second batch of rats was divided into four groups: Sham + LV‐NC, SCI + LV‐NC, SCI + LV‐MDK, and Sham + LV‐MDK, with 18 rats in each group. As described above, after successful anesthesia, rats were injected with LV‐NC or LV‐MDK as follows: Using the index finger of the dominant hand to palpate the lumbar vertebrae to locate the spinal midline, while the non‐dominant hand was used to elevate the rat to create a gap between the vertebrae for needle insertion. A microsyringe needle was inserted between two lumbar vertebrae at a 70° angle along the spinal midline with the bevel facing the rat's head. Once the needle contacted the bone, the angle was adjusted to 30°, and the needle was gently advanced into the subarachnoid space. Then, 10 μL of lentiviral suspension was injected by slowly pressing the plunger. After injection, the needle was rotated 180° once or twice and then withdrawn from the spine. Three days later, all four groups of rats underwent SCI surgery as previously described. On day 7 post‐SCI, six rats from each group were used for validation of MDK overexpression, western blotting, and immunofluorescence analysis. On day 42 post‐injury, all rats were euthanized, and spinal cord tissues from segments T9–T11 were collected for further analysis.

### 2.5 Lentiviral Vector Construction

Lentiviruses containing Midine (NM_030859.3) were constructed and synthesized by GenePharma lnc (Shanghai, China). In addition, a negative control gene lentiviral vector (lenti‐control) was generated using a nontargeted cDNA sequence. The final lentiviral vector concentration was 1 × 109 TU/mL.

### 2.6 Behavioral Test

Motor function recovery was assessed using the Basso, Beattie, and Bresnahan (BBB) scale and CatWalk gait analysis. All behavioral tests were conducted on days 1, 3, 5, 7, 10, 14, 21, 28, 35, and 42 post‐SCI surgery by two independent investigators who were blinded to the study. The BBB test was performed in an open field (1 × 1 m), and all rats were acclimated to the field before the formal testing. The BBB score ranges from 0 to 21, with higher scores indicating better motor function recovery in SCI rats.

On day 42 post‐SCI, gait analysis was performed using the CatWalk XT automated quantitative gait analysis system (Noldus, Wageningen, Netherlands) in each group of rats (*n* = 6 per group). During the gait analysis, the testing environment was kept dark to avoid interference from external light on the footprints. Gait parameters were automatically analyzed using CatWalk XT software. Subsequently, all parameters were carefully inspected and manually corrected frame by frame to ensure the accuracy of the results.

### 2.7 MEP

An electromyography evoked potential system (Hai Shen, Shanghai, China) was used to assess motor evoked potentials (MEPs) at 42 days after spinal cord injury (SCI) surgery. As previously described, rats were anesthetized by intraperitoneal injection of sodium pentobarbital. For MEP recording, the stimulating electrode was inserted through the nostril to contact the dura mater, the recording electrode was placed in the belly of the gastrocnemius muscle, the reference electrode was positioned between the fourth and fifth digits, and the ground electrode was inserted into the tail. Three stable MEPs were recorded at 1‐min intervals.

### 2.8 H&E Staining

Spinal cord tissues from T9 to T11 were collected and further fixed in 4% paraformaldehyde for 24 h. The tissues were then embedded in paraffin and sectioned into 5 μm slices. For H&E staining, the sections were stained with hematoxylin and eosin (H&E) in accordance with the supplier's specifications for H&E staining (C0105, Beyotime, China).

### 2.9 Western Blotting

The T9‐T11 spinal cord tissue samples were collected and homogenized in radioimmunoprecipitation assay (RIPA) lysis buffer. Then, the supernatants of tissue lysates were collected by 15 min centrifugation (12,000*g*; 4°C). Protein concentrations were determined using a bicinchoninic acid (BCA) protein assay. Equal amounts of total protein (30 μg) were separated by 10%–15% sodium dodecyl sulfate‐polyacrylamide gel electrophoresis (SDS‐PAGE) and transferred onto polyvinylidene difluoride (PVDF) membranes (Millipore, Billerica, MA, USA). The membranes were blocked with a mixture of 5% non‐fat milk and 5% bovine serum albumin (BSA) at room temperature for 1 h, followed by overnight incubation at 4°C with primary antibodies. The following primary antibodies were used: MDK (1:500, ABclonal, Cat# A025), LRP‐1 (1:10000, abcam, Cat# ab9254), Rac1 (1:1000, proteintech, Cat# 66122–1‐IG), p‐AKT (1:1000, CellSignaling Technology, Cat# 9271S), AKT (1:1000, CellSignaling Technology, Cat# 9272S), p‐mTOR (1:1000, CellSignaling Technology, Cat# #2971), mTOR (1:1000, CellSignaling Technology, Cat# #2983), BDNF (1:100, Santa Cruz Biotechnology, Cat# sc‐546), IL‐1β (1:1000, wanleibio, Cat# WL02257), TNF‐α (1:1000, ABclonal, Cat# A23264), IL‐6 (1:1000, wanleibio, Cat# WL02841), IL‐10 (1:1000, proteintech, Cat# 60269–1‐IG), and TGF‐β (1:1000, CellSignaling Technology, Cat# 3711). After washing with TBST, the membranes were incubated with horseradish peroxidase (HRP)‐conjugated secondary antibodies at room temperature for 2 h. Protein bands were visualized using enhanced chemiluminescence (ECL). The level of protein expression was quantified by Image J software.

### 2.10 RNA Extraction and Real‐Time Polymerase Chain Reaction

Total RNA from cells (*n* = 3 per group) was extracted using TRIzol reagent (Molecular Research Center, Cincinnati, OH, USA) according to the manufacturer's instructions. RNA was reverse transcribed into cDNA using a reverse transcription system (Promega, Madison, WI, USA). RT‐PCR was performed on an ABI 7900 PCR detection system using SYBR Green PCR Master Mix (Applied Biosystems, Foster City, CA, USA). Parallel amplification of the GAPDH gene was used to normalize the gene expression. The relative expression level of target mRNA was calculated using the ^ΔΔ^Ct method PCR primer sequences used in the study are listed in Table 1.

**TABLE 1:** Real‐time PCR primers used in the study.

Gene	Forward primer 5′‐3′	Reverse primer 5′‐3′
TNF‐α	CAGGCGGTGCCTATGTCTC	CGATCACCCCGAAGTTCAGTAG
IL‐1β	GCAACTGTTCCTGAACTCAACT	ATCTTTTGGGGTCCGTCAACT
IL‐6	CTGCAAGAGACTTCCATCCAG	AGTGGTATAGACAGGTCTGTTGG
β‐actin	CATCCGTAAAGACCTCTATGCCAAC	ATGGAGCCACCGATCCACA

### 2.11 Immunofluorescence and Imaging

Spinal cord tissues from the T9 to T11 segments were removed and postfixed with a 4% paraformaldehyde solution for 24 h at room temperature and a 30% sucrose solution for 24 h at 4°C. The samples were frozen and sectioned to a thickness of 5 μm using a cryostat (CM1900 UV; Leica Microsystems GmbH, Wetzlar, Germany) for subsequent immunofluorescence analysis. Briefly, the sections were blocked for 1 h by PBS with 10% donkey serum albumin and 0.3% Triton X‐100 at room temperature, and then the sections were washed with PBS and incubated overnight with primary antibodies at 4°C. The following primary antibodies were used: MDK (1:200, ABclonal, Cat# A025), LRP‐1 (1:100, abcam, Cat# ab9254), IBA‐1 (1:200, abcam, Cat# ab283319), GFAP (1:400, CellSignaling Technology, Cat# #3670), NeuN (1:400, abcam, Cat# ab104224), Cleaved‐Caspase3 (1:200, Affinity, Cat# AF7022), iNOS (1:200, Abmart, Cat# TTA0199), and CD206 (1:400, proteintech, Cat# 118704‐1‐AP). Subsequently, they were washed and incubated for 2 h at room temperature with appropriate secondary antibodies. The following secondary antibodies were used: Rabbit, and Mouse. Finally, images were acquired using a Zeiss LSM 800 confocal microscope and processed with Zeiss imaging software.

### 2.12 Extraction and Culture of Primary Neurons of Fetal Rats

In brief, the method for primary cortical neuron isolation was performed as previously described [15]. Pregnant rats at embryonic day 15–17 were euthanized by cervical dislocation, and the embryos were removed and briefly rinsed in chilled 75% ethanol. Under a dissection microscope, the fetal scalp was peeled open with forceps in pre‐cooled HBSS buffer (Thermo Fisher Scientific, Waltham, MA, USA), followed by a midline incision of the skull. The skull was opened to expose the brain, and the cerebral hemispheres were isolated to collect the cerebral cortex. The dissected cortices were minced in pre‐cooled enzymatic digestion solution and transferred into a 15 mL centrifuge tube for digestion at 37 C for 12 min. Digestion was terminated by adding 20% fetal bovine serum (FBS), and the cell suspension was passed through a 40 μm cell strainer, then centrifuged at 1000 rpm for 8 min. The resulting cell pellet was resuspended in Neurobasal Medium (Thermo Fisher Scientific, Waltham, MA, USA) supplemented with B27 (Thermo Fisher Scientific), GlutaMAX‐I (Thermo Fisher Scientific), penicillin–streptomycin (Thermo Fisher Scientific), HEPES, and horse serum. Cells were seeded onto dishes pre‐coated with poly‐D‐lysine. After 16 h, the medium was replaced with fresh Neurobasal Medium containing B27, GlutaMAX‐I, and penicillin–streptomycin. Cultures were maintained at 37 C in a humidified atmosphere of 5% CO_2_ and 95% air. Half of the medium was replaced every 2 days. Apoptosis was induced in primary neurons on day 6 after planting.

### 2.13 Cell Culture and Treatment

BV‐2 cells (EK‐Bioscience, Shanghai, China, Cat# CC‐Y2022, RRID: CVCL_5I31) were cultured in Dulbecco's Modified Eagle Medium (DMEM; Fude), supplemented with 10% (v/v) fetal bovine serum (FBS), 100 U/mL penicillin, and 100 U/mL streptomycin, in a humidified atmosphere containing 5% CO_2_ at 37°C. Cells were seeded into 12‐well plates and confocal dishes for subsequent experiments.

Lipopolysaccharide (LPS; 1 μg/mL) and recombinant human midkine (rhMDK; 200 ng/mL) were co‐administered to BV‐2 cells and incubated for 12 h. For experiments involving RAP (50 μM), RAP was added 30 min before LPS and rhMDK treatment, and the remaining procedures were consistent with the above.

### 2.14 Microglial Efferocytosis Assay

BV2 cells were seeded onto confocal dishes. Primary cortical neurons were isolated from rat embryos and cultured for 7 days in vitro before use, resuspended in 3 mL PBS, and transferred into culture dishes. To induce apoptosis, the neurons were exposed to ultraviolet (UV) light for 30 min. Subsequently, BCECF fluorescent dye (1:1000 dilution) was added, and the cells were incubated for 30 min at 37 C in a humidified atmosphere of 5% CO_2_ and 95% air, protected from light. After staining, the neurons were centrifuged at 1200 rpm for 5 min to remove excess dye and resuspended in PBS. Approximately 100 μL of the labeled apoptotic neurons were added to each confocal dish containing pre‐treated BV2 cells and co‐incubated for 15 min under the same culture conditions. After incubation, cells were washed three times with pre‐cooled PBS to remove non‐phagocytosed apoptotic neurons. The samples were then imaged using a confocal microscope, and the percentage of phagocytic (positive) cells was quantified.

### 2.15 Statistical Analysis

Data are expressed as mean ± standard error of the mean (SEM). For measurements involving multiple groups across different time points (e.g., BBB scores), two‐way analysis of variance (ANOVA) was employed. Comparisons among more than two groups were assessed using one‐way ANOVA followed by Tukey's post hoc test. Statistical analyses were performed using Prism 10.1.1 (GraphPad Software Inc., USA). A *p*‐value less than 0.05 was considered statistically significant.

## 3 Results

### 3.1 Transcriptomic Profiling Reveals Inflammatory and Cell Death Pathways Activated After SCI

**FIGURE 1:**
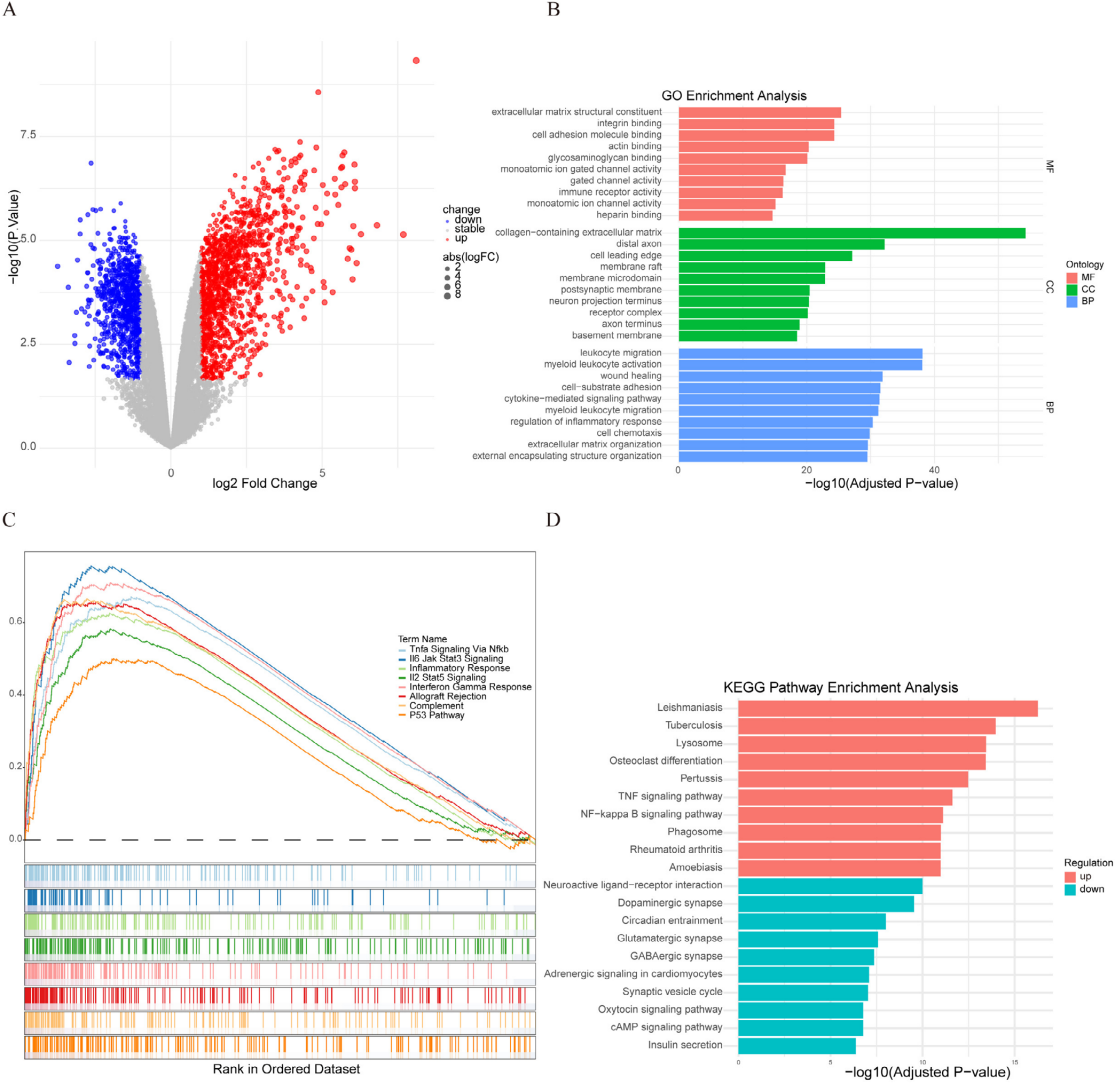
Transcriptomic profiling of spinal cord injury (SCI)‐induced gene expression alterations. (A) Volcano plot showing differentially expressed genes (DEGs) in spinal cord tissue following SCI (GSE93561 dataset). Genes meeting significance thresholds (adjusted *p* < 0.05, |log2FC| > 1) are highlighted in red (upregulated) and blue (downregulated). Gray dots represent non‐significant genes. (B) Gene Ontology (GO) enrichment analysis of SCI‐associated DEGs, the chord diagram displays significantly enriched terms (FDR < 0.05) in biological processes (top), cellular components (middle), and molecular functions (bottom), with connecting ribbons representing gene‐term associations. (C) Gene Set Enrichment Analysis (GSEA) of hallmark gene sets in this dataset, highlighting pathways associated with SCI. (D) Kyoto Encyclopedia of Genes and Genomes (KEGG) pathway analysis of DEGs in this dataset, highlighting pathways altered after SCI.

To explore the molecular alterations following SCI, we analyzed a publicly available microarray dataset (GSE93561) and selected six samples from the Uninjured and SCI groups (GSM2454718–GSM2454723). After quality control and differential gene expression analysis, we identified 1470 upregulated and 894 downregulated genes in the SCI group compared to the Uninjured group (Figure 1A). Subsequently, GO, GSEA, and KEGG analyses were performed to assess the functional significance of the differentially expressed genes (DEGs) (Figure 1B–D). GO enrichment analysis was performed from three major aspects: molecular function (MF), cellular component (CC), and biological process (BP), to help elucidate changes in biological functions and regulatory mechanisms following SCI (Figure 1B). In the MF category, DEGs were mainly involved in extracellular matrix structural constituent, actin binding, cell adhesion molecule binding, and positive regulation of immune receptor activity. The CC analysis revealed significant enrichment of distal axon, membrane microdomain, neuron projection terminus, receptor complex, and axon terminus. BP analysis revealed enrichment in leukocyte migration, myeloid leukocyte activation, wound healing, and regulation of inflammatory response. The results indicated that inflammatory responses and cell death pathways were strongly activated following SCI. Furthermore, KEGG pathway analysis indicated upregulation of Lysosome, TNF signaling pathway, NF‐kappa B signaling pathway, and Phagosome pathways in the SCI group (Figure 1D). Collectively, these findings highlight prominent activation of inflammation‐, apoptosis‐, and immune microenvironment‐related pathways during the subacute phase of SCI.

### 3.2 Single‐Cell Transcriptomics Reveals Altered Cell–Cell and Ligand–Receptor Communication Following SCI

**FIGURE 2:**
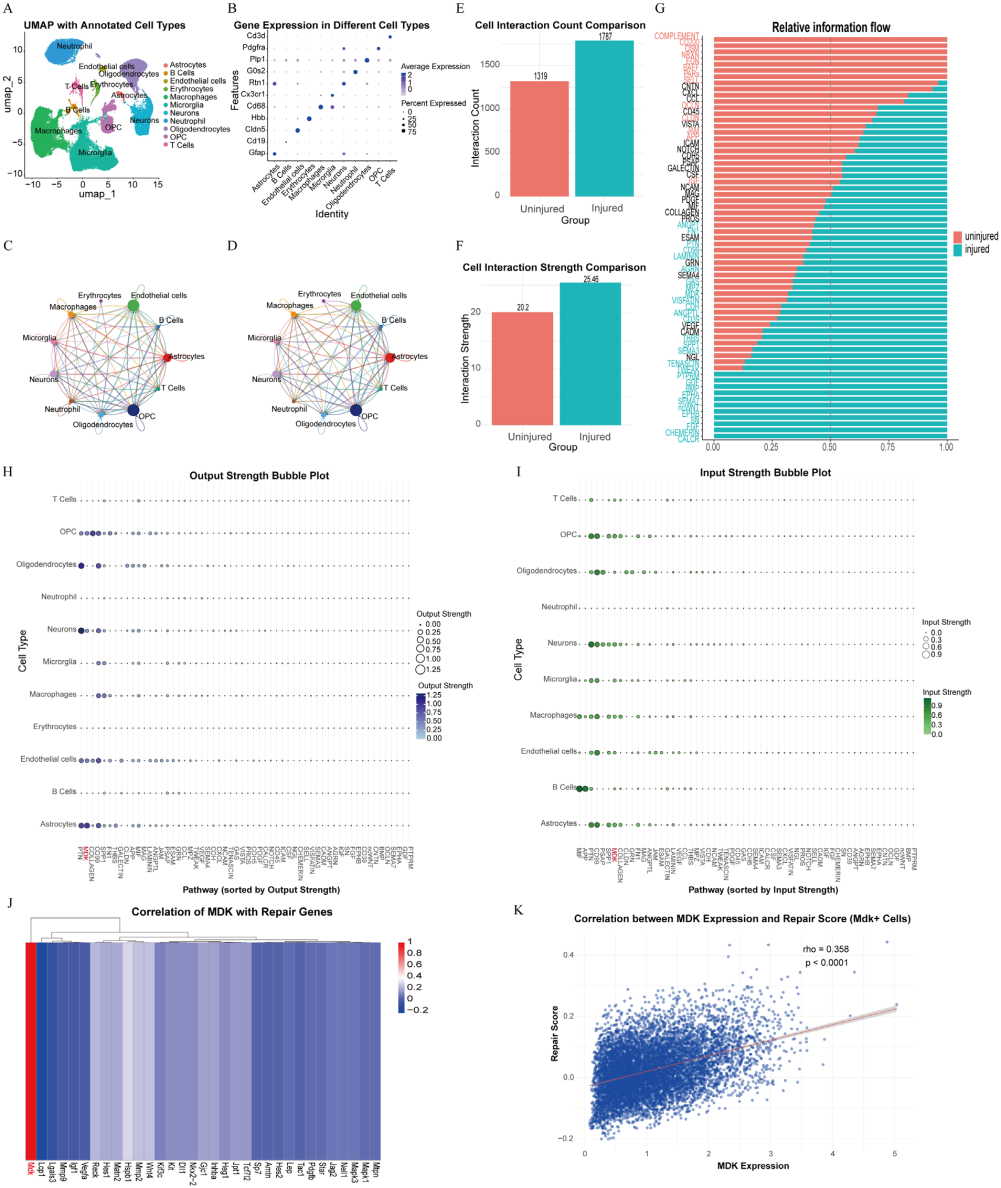
Single‐cell transcriptomic analysis of intercellular communication dynamics at 7 days post‐SCI in the GSE213240 dataset. (A) Uniform Manifold Approximation and Projection (UMAP) representation of cellular heterogeneity in the injured spinal cord, with distinct colors denoting identified cell clusters. (B) Bubble chart shows the expression of marker genes in each cell cluster. The circle size represents the proportion of gene expression in the cell cluster. Color intensity represents average gene expression. (C, D) Quantitative assessment of cell–cell communication alterations: Changes in the number of interactions after SCI. Changes in the strength of interactions after SCI. (E, F) Comparative analysis of intercellular signaling: (E) Bar graph showing the differential number of interactions between the control and SCI group. (F) Bar graph showing the differential strength of interactions between the control and SCI group. (G) Relative information flow changes after SCI indicate upregulation of the MDK signaling pathway. (H–I) Ligand‐receptor interaction signatures: (H) Bubble plot displaying ligand–receptor interactions in the Injured group after SCI, ordered by output signaling strength from left (strong) to right (weak). (I) Bubble plot displaying ligand–receptor interactions in the Injured group after SCI, ordered by input signaling strength from left (strong) to right (weak). (J) Correlation analysis showed that the MDK gene is highly correlated with the rat neurorepair gene set from the Regeneration Roadmap database. (K) Scatter plot with regression line showing positive correlation between MDK^+^ cell abundance and tissue repair scores across samples.

To elucidate intercellular signaling dynamics after spinal cord injury (SCI), we analyzed a publicly available single‐cell RNA sequencing (scRNA‐seq) dataset (GSE213240) comprising spinal cord samples from eight rats with varying degrees of injury. After quality control and batch effect correction, 68,658 cells were retained for downstream analysis. Dimensionality reduction using UMAP revealed 11 distinct cell clusters (Figure 2A), which were annotated based on canonical marker genes as astrocytes (Gfap), B cells (Cd19), endothelial cells (Cldn5), erythrocytes (Hbb), macrophages (Cd68), microglia (Cx3cr1), neurons (Rtn1), neutrophils (G0s2), oligodendrocytes (Plp1), OPCs (Pdgfra), and T cells (Cd3d) (Figure 2B). We used the CellChat R package to analyze changes in SCI‐induced intercellular communication. Both the number and strength of inferred cell–cell communications were significantly increased following SCI (Figure 2C–F). Ligand–receptor analysis further revealed substantial changes in intercellular signaling patterns, with significant enrichment of the MDK and Pleiotrophin (PTN) signaling pathways in the SCI group (Figure 2G–I). To validate the relevance of MDK in nerve repair, we queried the Regeneration Roadmap database provided by the National Genomics Data Center using the keywords “Rat” and “nerve regeneration” [16]. We found that MDK was identified within the curated list of nerve injury‐ and repair‐associated genes (Figure 2J). Correlation between MDK‐expressing cells in the scRNA‐seq dataset and the nerve repair‐related gene set confirmed a strong association between MDK expression and nerve regeneration processes (Figure 2K).

### 3.3 Temporal Expression and Spatial Location of MDK Following SCI

**FIGURE 3:**
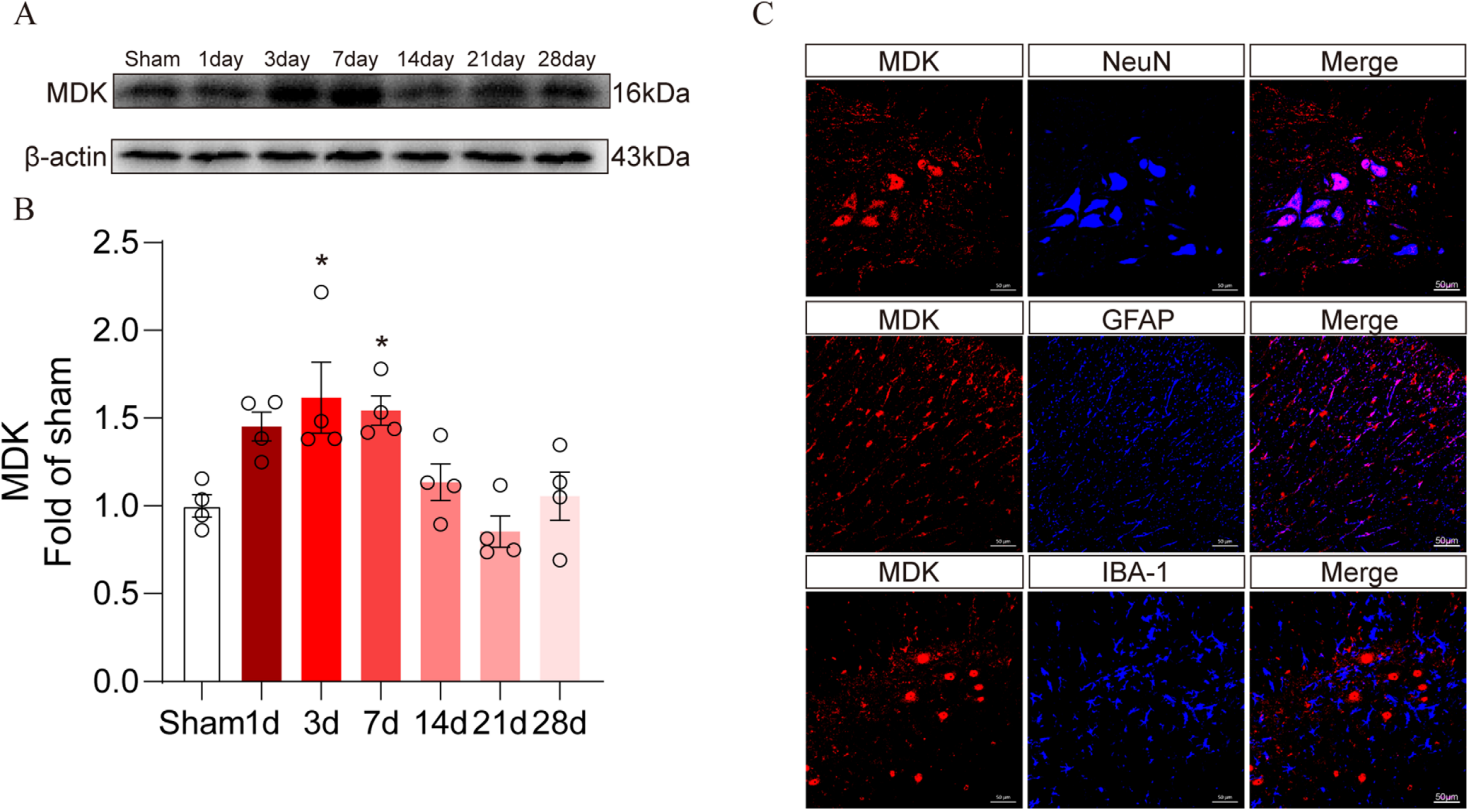
Temporal expression and Spatial location of MDK Following SCI. (A) Western blot analysis of MDK expression in the spinal cords at different stages after SCI. (B) Quantitative analysis of the relative MDK level (normalized to Sham group) as shown in (A) (*n* = 4 per group). All data are presented as the mean ± SEM. *p < 0.05, as determined by one‐way ANOVA. (C) Expression of MDK in neurons, astrocytes, and microglia identified by immunofluorescence staining for NeuN, GFAP, and IBA‐1; scale bar = 50 μm (*n* = 3).

MDK is known to be rapidly induced in response to SCI. To investigate its temporal expression following SCI, we first examined MDK protein levels at the lesion site using Western blotting. MDK expression was significantly upregulated in the early stages post‐injury, with a marked increase observed at day 3, peaking at day 7 before declining to near‐baseline levels thereafter (Figure 3A). Immunofluorescence staining revealed that MDK was primarily expressed in NeuN‐positive neurons and GFAP‐positive astrocytes. Notably, MDK exhibited a spatial distribution surrounding Iba1‐positive microglia, suggesting a potential role in microglia‐associated signaling during SCI repair (Figure 3B).

### 3.4 MDK Overexpression Promotes Functional Recovery and Spinal Tissue Repair Following SCI

**FIGURE 4:**
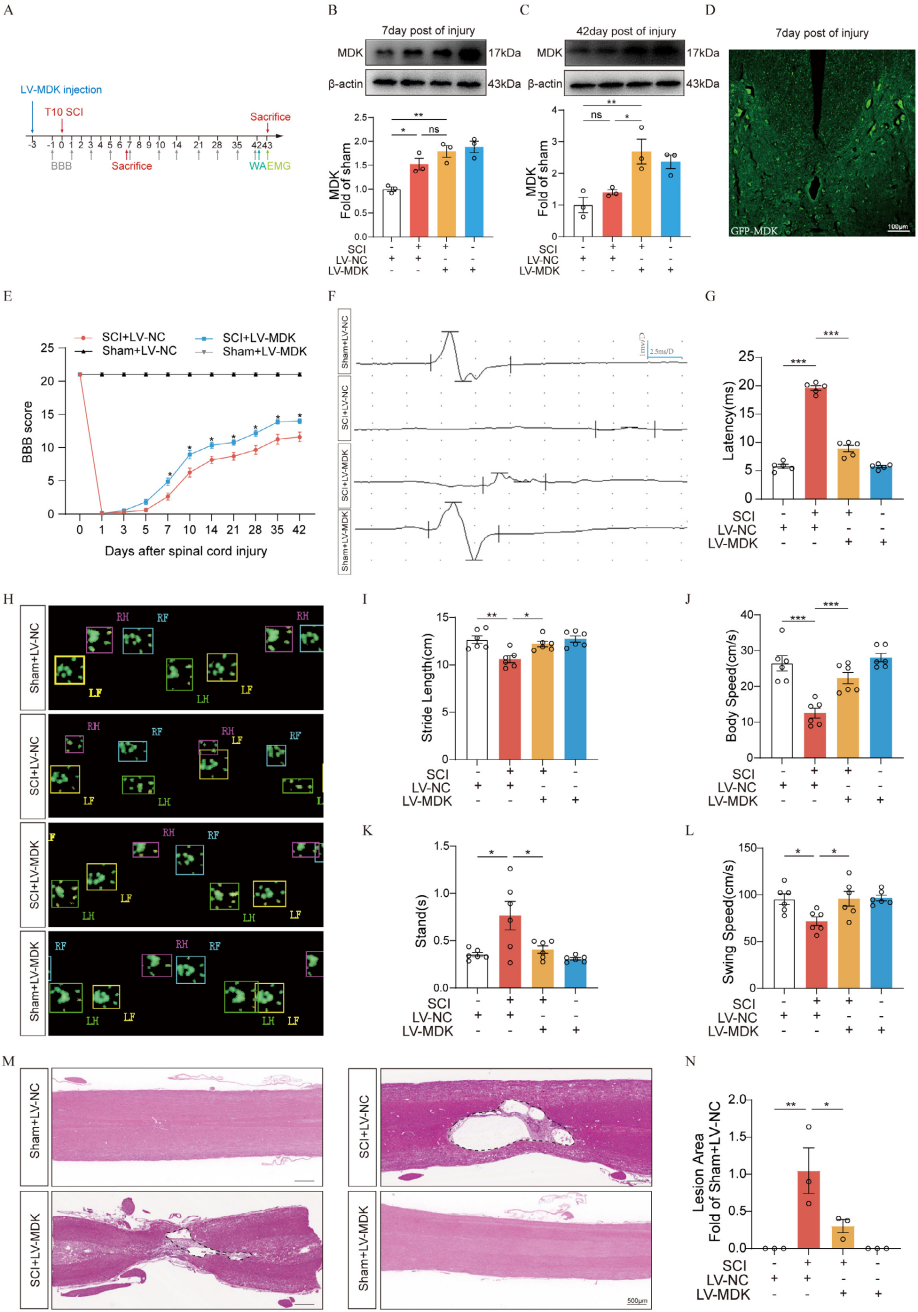
MDK overexpression promotes motor function recovery and spinal cord tissue repair in rats following SCI. (A) Timeline diagram of spinal cord injury, LV‐MDK treatment, and experimental analysis. (B) Western blot analysis showing the expression of MDK at the injury site on day 7 post‐SCI (*n* = 3). (C) Western blot analysis showing the expression of MDK at the injury site on day 42 post‐SCI (*n* = 3). (D) Distribution of lentivirus (GFP+) expression in the spinal cord on day 7 post‐SCI. Scale bar = 100 μm. Green fluorescence indicates a tissue transfected with LV. (E) The BBB scores in each group (*n* = 10) values are expressed as the mean ± SEM.*p < 0.05, as determined by two‐way analysis of variance (ANOVA). (F) Representative images of motor‐evoked potential (MEP) in each group (*n* = 5). (G) Quantification analysis of the latency of MEP. (H) The representative images of footprint intensity in each group (*n* = 6). (L) Quantification analysis of the CatWalk of gait analysis parameters in each group. SCI + LV‐MDK group displayed significant decreases in the CatWalk of gait analysis parameters after SCI at the 42 day: (I) Stride Length, (J) Body Speed, (L) Stand, (M) Swing Speed. (M) Representative HE staining images in longitudinal sections of spinal cord from each group (*n* = 3). Scale bar = 500 μm. (N) Quantification of the percentage of injured area. Values are expressed as the mean ± SEM. *p < 0.05, **p < 0.01, ***p < 0.001, and ****p < 0.0001, as determined by one‐way ANOVA.

To investigate the therapeutic role of MDK following SCI, we employed a lentiviral vector encoding MDK (LV‐MDK) into the spinal cord parenchyma to upregulate MDK expression (Figure 4A). Western blotting analysis confirmed significantly increased MDK expression at the injury site in the LV‐MDK group compared to controls at both 7 and 42 days post‐injection (Figure 4B,C). Immunofluorescence further confirmed successful viral transduction in the spinal cord (Figure 4D). Next, we evaluated the therapeutic effects of MDK overexpression following SCI. Functional recovery was assessed using the BBB locomotor rating scale (Figure 4E). Although no significant differences in BBB scores were observed between the SCI + LV‐NC and SCI + LV‐MDK groups on days 3 and 5 post‐injury, the LV‐MDK group exhibited significantly higher scores from day 7 onward, with sustained improvement maintained through day 42 (Figure 4E). To further validate the impact of LV‐MDK on functional recovery, MEP and gait analysis were conducted at 42 days post‐injury (Figure 4F–L). LV‐MDK treatment significantly increased MEP amplitude and reduced MEP latency (Figure 4F,G). Gait analysis revealed improved hindlimb footprint integrity and size in the LV‐MDK group, along with significant improvements in multiple weight‐independent gait parameters (Figure 4H–L). In addition to functional gains, histological assessment by hematoxylin–eosin (HE) staining demonstrated a marked reduction in lesion area in the LV‐MDK group at 42 days post‐injury (Figure 4M,N). Collectively, these findings indicate that MDK upregulation promotes both structural repair and motor function recovery following SCI in rats.

### 3.5 Overexpression of MDK Reduces Neuronal Apoptosis and Promotes Neuroprotection After SCI

**FIGURE 5:**
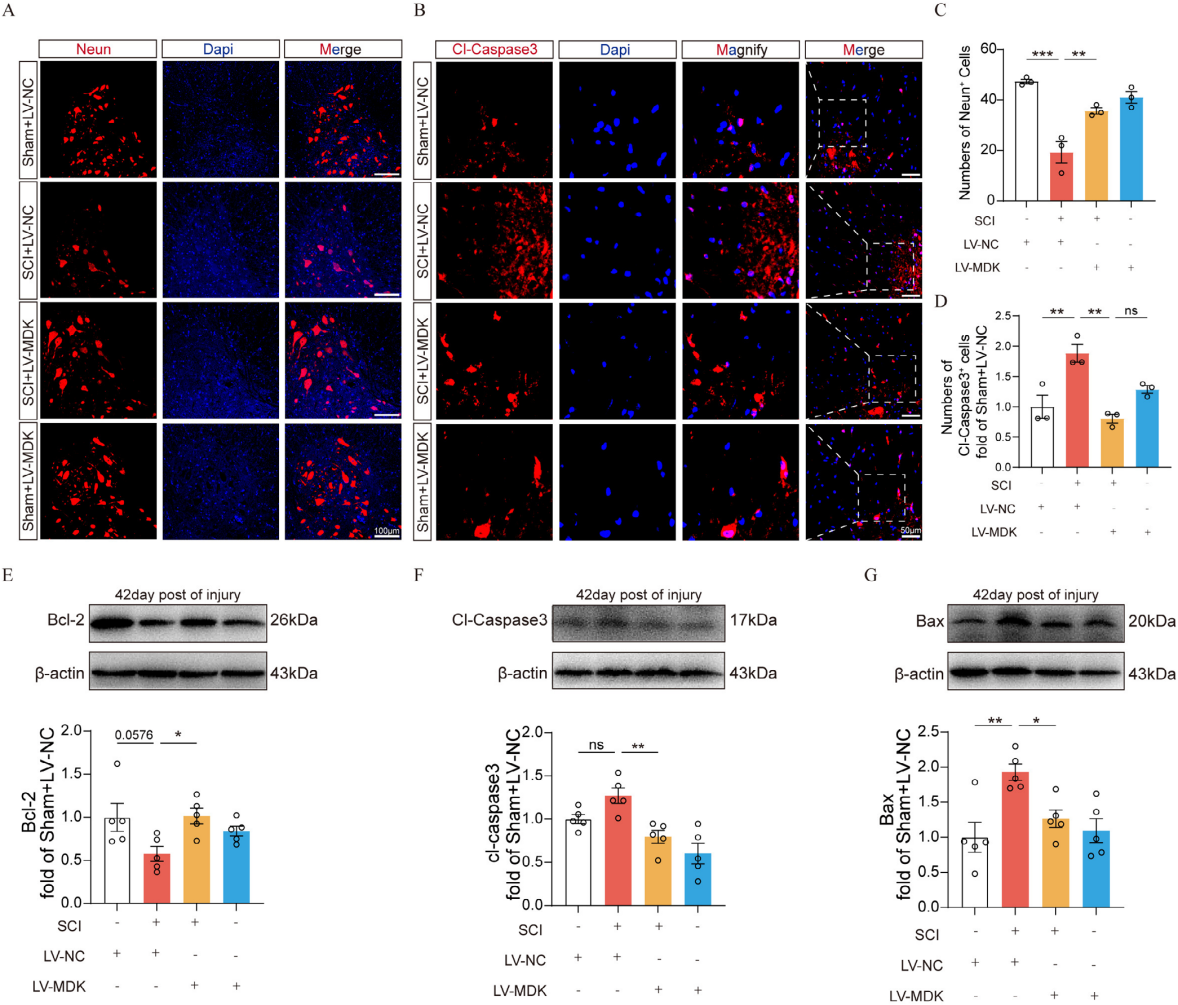
MDK overexpression inhibited SCI‐induced apoptosis. (A) Representative immunofluorescence staining images of NeuN (red) in the anterior horn of the spinal cord from each group at 42 days after SCI (*n* = 3). Nuclei were counterstained with DAPI (blue). Scale bar = 100 μm. (B) Representative immunofluorescence staining images of Cl‐Caspase3 (red) in the anterior horn of the spinal cord from each group at 7 days after SCI. (*n* = 3). Nuclei were counterstained with DAPI (blue). Scale bar = 50 μm. (C) Quantification of the number of NeuN cells in (A). (D) Quantification of the number of Cl‐Caspase3^+^ cells in (B). (E–G) Representative images of Western blotting and quantification analysis of Bcl‐2, Cleaved‐Caspase3, Bax, and β‐actin in each group (*n* = 4), values are expressed as the mean ± SEM. *p < 0.05, **p < 0.01, and ***p < 0.001 as determined by one‐way ANOVA.

Neuronal survival is essential for functional recovery following SCI. To investigate the neuroprotective effect of MDK expression, we performed NeuN immunofluorescence staining. At 42 days post‐SCI, a marked reduction in NeuN‐positive cells was observed in the spinal cord anterior horn. However, LV‐MDK treatment significantly preserved NeuN‐positive cells compared to controls (Figure 5A,C). To evaluate apoptosis, we respectively conducted TUNEL and Cleaved‐Caspase‐3 immunofluorescence staining. At 7 days post‐injury, apoptotic cells were markedly increased in the injured spinal cord, while the SCI + LV‐MDK group showed a significant reduction in Cleaved‐Caspase‐3‐positive and TUNEL‐positive cells compared to the SCI + LV‐NC group (Figure 5B,D). Western blot analysis further confirmed the anti‐apoptotic effect of MDK overexpression, demonstrating decreased expression of Cleaved‐Caspase‐3 and Bax, and increased expression of the anti‐apoptotic protein Bcl‐2 at the lesion site (Figure 5E–G). Collectively, these data suggest that MDK overexpression mitigates neuronal loss and reduces neuronal apoptosis, thereby exerting a neuroprotective effect in SCI.

### 3.6 MDK Overexpression Attenuates Neuroinflammation and Promotes M2 Polarization of Microglia Following SCI

**FIGURE 6:**
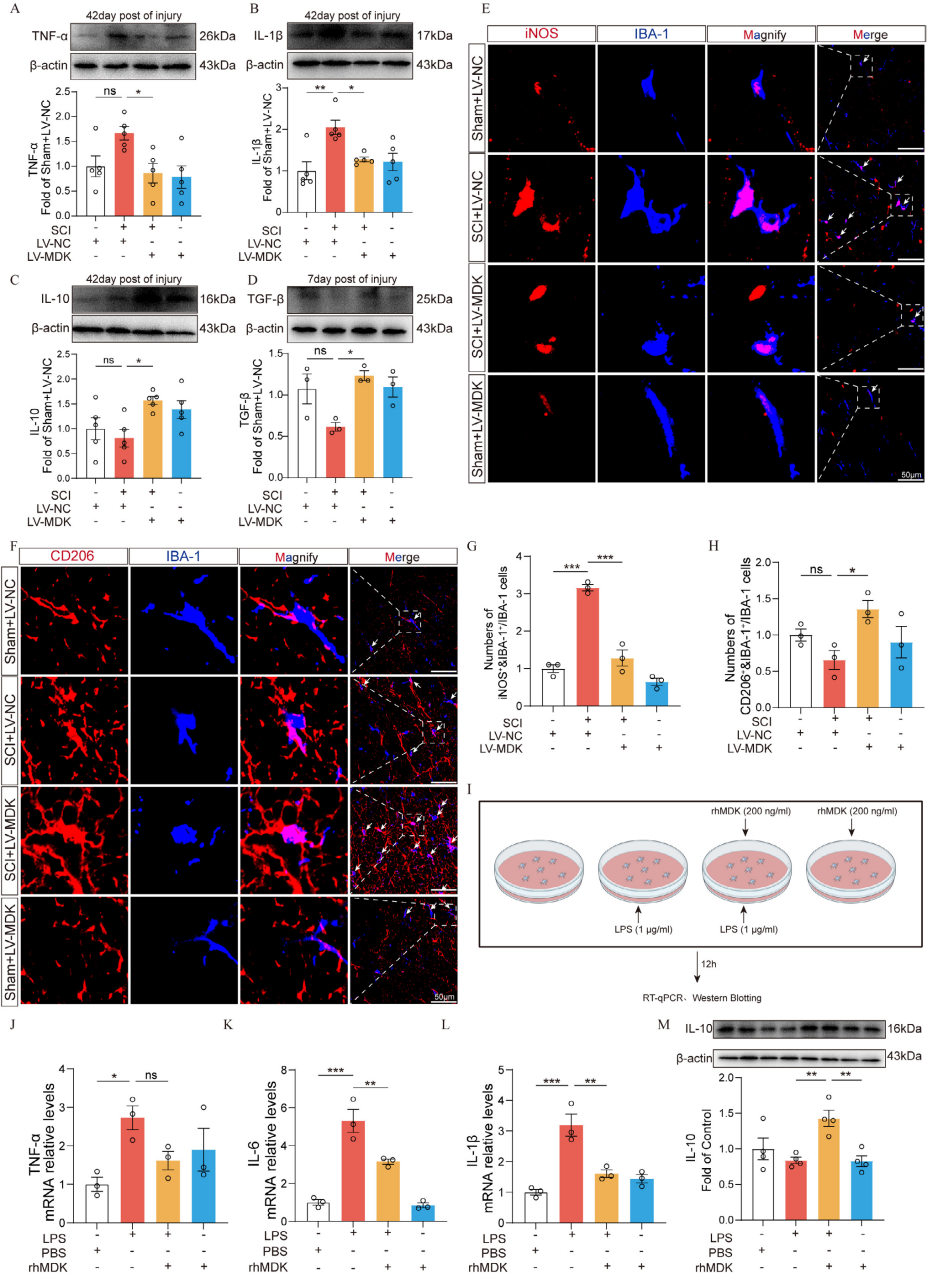
MDK overexpression drives microglial polarization toward an anti‐inflammatory phenotype and attenuates neuroinflammation after SCI. (A–C) Representative images of Western blotting and quantification analysis of TNF‐α (A), IL‐1β (B), IL‐10 (C), β‐actin in each group (*n* = 5). (D) Representative images of Western blotting and quantification analysis of TGF‐β, β‐actin in each group (*n* = 3). (E) Representative iNOS and IBA‐1 immunofluorescence staining images in the injured spinal cord. Scale bar: 50 μm. Arrows indicate iNOS and IBA‐1 (iNOS: Red; IBA‐1: Blue) cells. (F) Representative CD206&IBA‐1 immunofluorescence staining images in the injured spinal cord. Scale bars: 50 μm. Arrows indicate CD206&IBA‐1 (CD206: Red; IBA‐1: Blue) cells. (G) Quantitative analysis of the numbers of iNOS^+^&IBA‐1^+^/IBA‐1^+^ cells. (H) Quantitative analysis of the numbers of CD206^+^&IBA‐1^+^/IBA‐1^+^ cells. (I) Schematic diagram of in vitro experiment. (J–L) Relative mRNA expression levels of TNF‐α, IL‐6, and IL‐1β in each group, as detected by qRT‐PCR. (M) Representative images of Western blotting and quantification analysis of IL‐10 and β‐actin, values are expressed as the mean ± SEM, *p < 0.05, **p < 0.01, ***p < 0.001, and ****p < 0.0001, as determined by one‐way ANOVA.

To evaluate the anti‐inflammatory effects of MDK, we examined pro‐inflammatory cytokine expression and microglial polarization in the spinal cord of SCI rats (Figure 6A–H). Western blot analysis revealed that MDK overexpression significantly suppressed SCI‐induced upregulation of pro‐inflammatory cytokines IL‐1β and TNF‐α, while enhancing the expression of anti‐inflammatory cytokines IL‐10 and TGF‐β (Figure 6A–D). Immunofluorescence staining further showed a marked reduction in iNOS‐positive (M1‐type) microglia and an increase in CD206‐positive (M2‐type) microglia in the SCI + LV‐MDK group compared to the SCI + LV‐NC group (Figure 6E–H), indicating a shift toward an anti‐inflammatory microglial phenotype. To validate these findings in vitro, we stimulated BV2 microglial cells with LPS and treated them with recombinant human MDK (rhMDK). qPCR results revealed that the rhMDK significantly reduced mRNA levels of IL‐1β, IL‐6, and TNF‐α (Figure 6J–L). Consistently, Western blotting showed increased IL‐10 protein expression following MDK treatment (Figure 6M). Collectively, these findings suggest that MDK overexpression attenuates neuroinflammation after SCI and promotes microglial polarization toward the anti‐inflammatory M2 phenotype.

### 3.7 MDK Overexpression Enhances Microglial Efferocytosis and Exerts Neuroprotective Effects in SCI Rats

**FIGURE 7:**
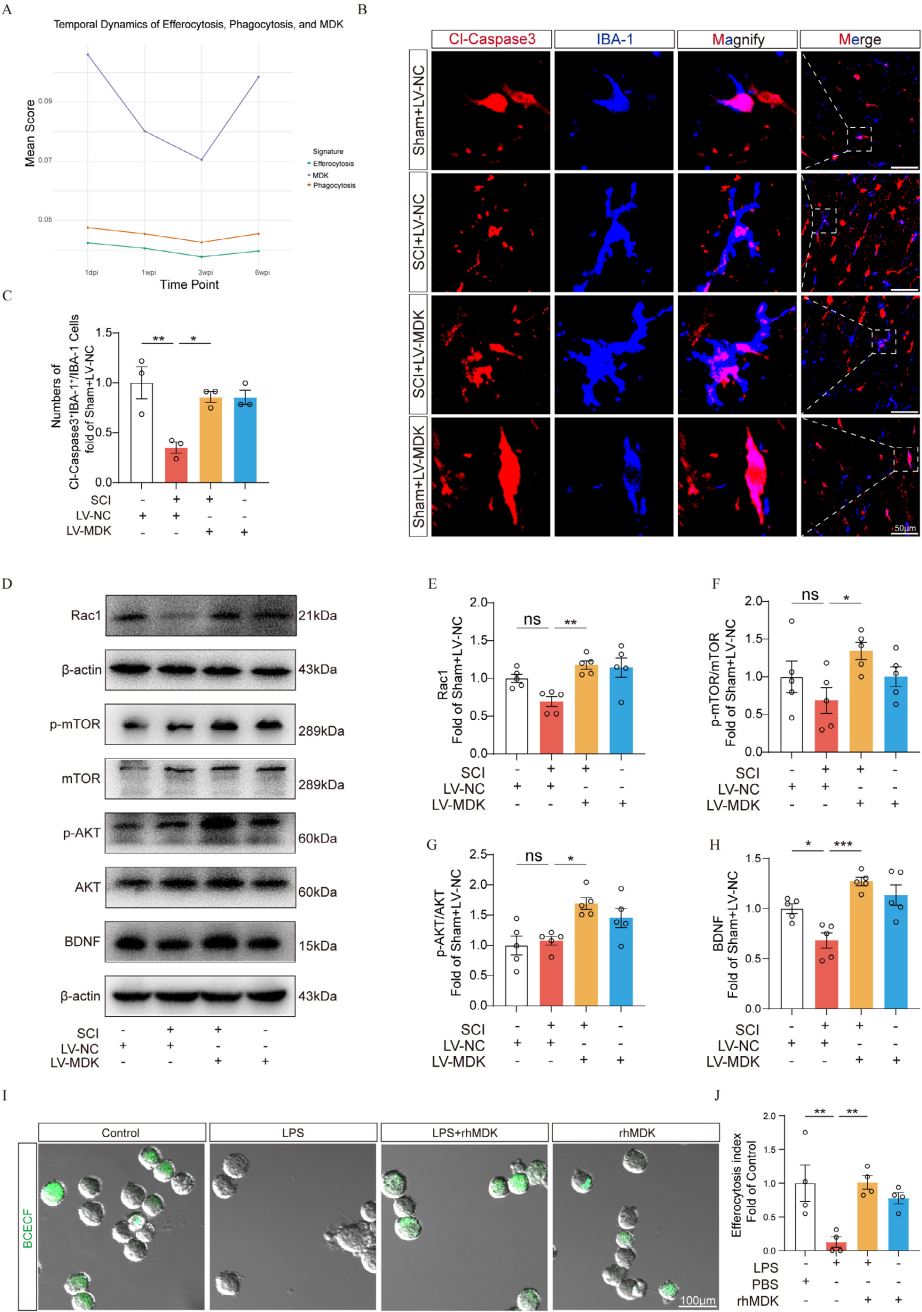
MDK overexpression enhances microglial efferocytosis and exerts neuroprotective effects. (A) Line plots generated by ggplot show the temporal expression patterns of MDK, efferocytosis‐related, and phagocytosis‐related genes in the GSE172167 dataset. (B) Representative Cl‐Caspase3 and IBA‐1 immunofluorescence staining images in the injured spinal cord. Scale bar: 50 μm. Arrows indicate iNOS and IBA‐1 (Cl‐Caspase3: Red; IBA‐1: Blue) cells. (C) Quantitative analysis of the numbers of Cl‐Caspase3^+^&IBA‐1^+^/IBA‐1^+^ cells. (D–H) Representative images of Western blotting and quantification analysis of Rac1, p‐AKT/AKT, p‐mTOR/mTOR, BDNF, and β‐actin in each group (*n* = 5). (I) BV2 was treated with LPS (1 μg/mL) for 12 h. Apoptotic primary neurons (green) were added to BV2, with or without rhMDK (200 ng/mL) for 12 h. Confocal microscopy was used to count the phagocytosis index. Scale bar = 100 μm. (J) Quantitative analysis of the number of Efferocytosis index.

Our previous studies established that spinal cord injury (SCI) significantly impairs microglial phagocytic function, leading to the accumulation of myelin debris that exacerbates neuroinflammatory responses [9]. Although defective efferocytosis is known to impair the clearance of apoptotic cells and exacerbate neuroinflammation [17], it remains unclear whether SCI directly disrupts this process. To investigate MDK's neuroprotective mechanisms, we analyzed scRNA‐seq data and found MDK expression temporally correlated with key efferocytosis‐ and phagocytosis‐related genes (Figure 7A), suggesting its regulatory role in these processes. To assess whether MDK overexpression enhances microglial phagocytic activity, we performed immunofluorescence co‐staining of IBA‐1 and Cleaved‐Caspase‐3 (Figure 7B,C). Notably, microglia in the SCI + LV‐MDK group exhibited significantly greater engulfment of apoptotic cells compared to the SCI + LV‐NC group (Figure 7B,C), indicating that MDK promotes efferocytosis in vivo. Next, we further explored the molecular mechanisms underlying MDK‐mediated efferocytosis and neuroprotection (Figure 7D–H). Western blot analysis revealed that MDK upregulation increased the expression of Rac1, a critical regulator of cytoskeletal reorganization during efferocytosis, as well as key signaling molecules p‐AKT/AKT and p‐mTOR/mTOR (Figure 7D–G). Additionally, elevated levels of brain‐derived neurotrophic factor (BDNF) suggested enhanced neurotrophic support in response to MDK overexpression (Figure 7D,H). These findings were further validated in vitro (Figure 7I,J). Immunofluorescence staining showed that LPS markedly impaired the ability of BV‐2 microglial cells to engulf apoptotic primary neurons, whereas MDK treatment effectively restored this efferocytic capacity. Collectively, these results demonstrate that MDK overexpression enhances the efferocytic capacity of microglia after SCI, thereby contributing to neuronal protection and repair.

### 3.8 MDK Facilitates Microglia Efferocytosis via LRP‐1

**FIGURE 8:**
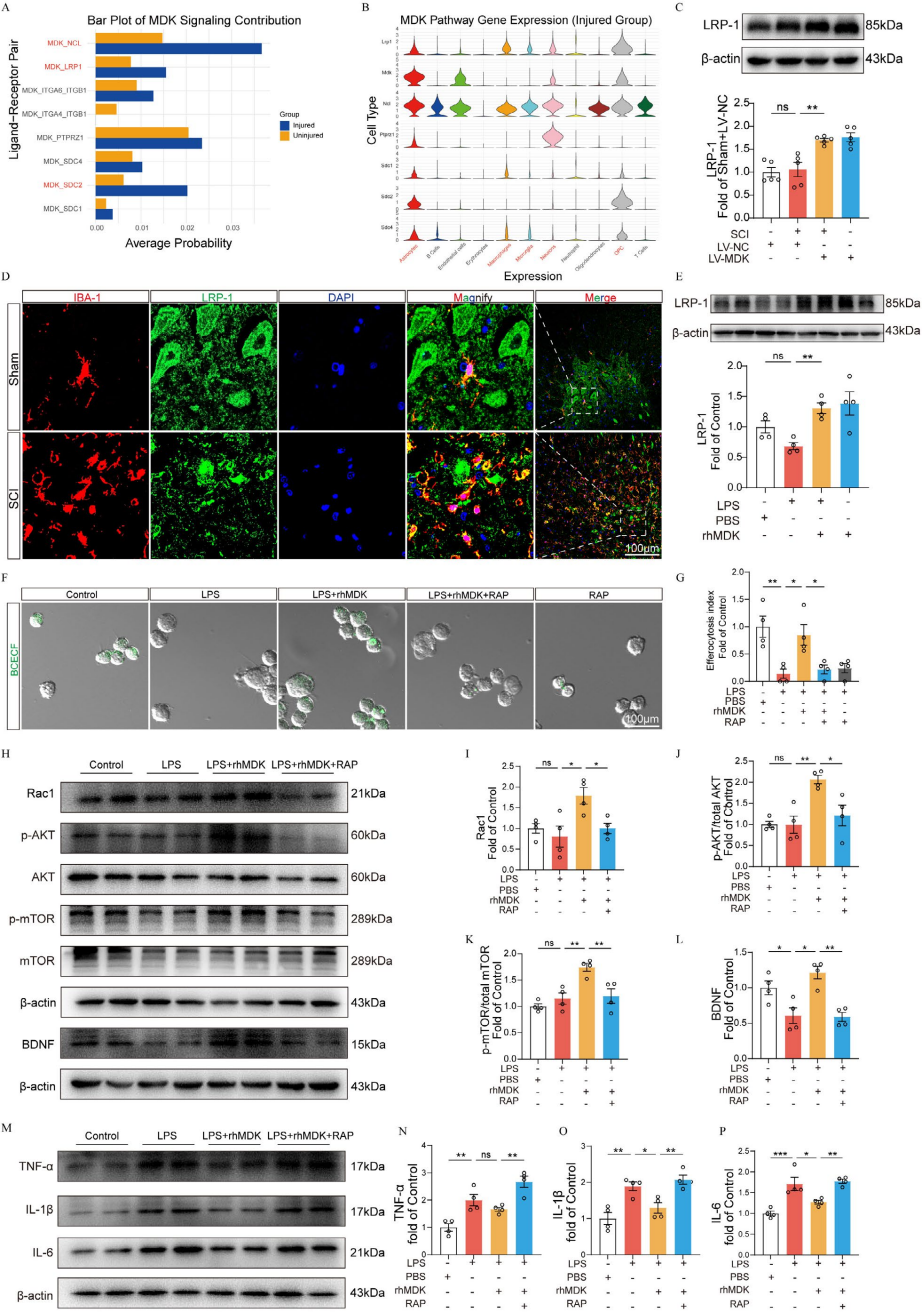
MDK enhances microglial efferocytosis through LRP‐1. (A) The bar graph shows changes in MDK receptor pathways before and after SCI in the GSE213240 dataset. (B) The violin plot shows the distribution of MDK and its receptors across cell types after SCI in the GSE213240 dataset. (C) Western blot analysis showing the expression of LRP‐1 at the injury site on day 42 post‐SCI (*n* = 5). (D) Representative immunofluorescence staining images of LRP‐1 in the spinal cord from each group at 28 days after SCI. Scale bar = 100 μm. (E) Representative images of Western blotting and quantification analysis of LRP‐1 in vitro experiment. (F) Representative immunofluorescence images of apoptotic neurons uptake by BV‐2 cells in each group (*n* = 4). Scale bar = 100 μm. (G) Quantification analysis of efferocytosis index within the BV‐2 cells. (H–L) Representative images of Western blotting and quantification analysis of Rac1, p‐AKT/AKT, p‐mTOR/mTOR, BDNF, and β‐actin in vitro experiment. (M–P) Representative images of Western blotting and quantification analysis of IL‐1β, IL‐6, TNF‐α, and β‐actin in vitro experiment.

To investigate the receptor‐mediated mechanism underlying MDK‐induced efferocytosis, we analyzed the single‐cell RNA sequencing dataset and found that the interactions between MDK and its known receptors—NCL, LRP1, and SDC2—were markedly increased following SCI (Figure 8A,B). Among these receptors, LRP1 emerged as the predominant MDK receptor [10], consistent with its established role as a key efferocytosis receptor in microglia [18]. Western blot analysis demonstrated that MDK overexpression (SCI + LV‐MDK group) significantly elevated LRP1 protein levels compared to SCI + LV‐NC group (Figure 8C). Immunofluorescence staining confirmed microglial expression of LRP1 in the injured spinal cord (Figure 8D), while in vitro experiments revealed that MDK treatment upregulated LRP1 in LPS‐stimulated BV‐2 cells (Figure 8E). To determine LRP1's functional involvement, we employed receptor‐associated protein (RAP), a specific LRP1 antagonist that competitively inhibits ligand binding. Remarkably, RAP treatment abolished MDK‐enhanced efferocytosis, as evidenced by significantly reduced apoptotic neuron engulfment by BV‐2 cells (Figure 8F,G), indicating that LRP1 blockade impairs MDK‐mediated efferocytosis. Consistent with this observation, Western blot analysis demonstrated that RAP treatment attenuated MDK‐induced upregulation of Rac1, as well as downstream effectors including p‐AKT/AKT, p‐mTOR/mTOR, and BDNF (Figure 8H,I). Collectively, these results suggest that MDK promotes microglial efferocytosis and activates neuroprotective signaling pathways via LRP1. Inhibition of LRP1 abolishes these effects, highlighting its essential role in MDK‐mediated modulation of microglial function.

## 4 Discussion

Following SCI, the inflammatory microenvironment and neuronal cell death hinder motor function recovery [19]. Therefore, alleviating neuroinflammation and clearing apoptotic cells at the injury site are critical for functional restoration [20]. This study identifies that MDK overexpression promotes microglial efferocytosis, reduces neuroinflammation and apoptosis, and enhances functional recovery. Mechanistically, MDK exerts its effects via the LRP‐1 receptor and activates the Rac1/AKT/mTOR/BDNF signaling pathway, highlighting the MDK–LRP‐1 axis as a promising therapeutic target for SCI.

### 4.1 Bioinformatics Analysis Reveals MDK as a Key Regulator in SCI Pathogenesis

The first key question addressed in this study is why we chose to focus on MDK. In this study, we reanalyzed previously published microarray sequencing data and found that the subacute phase of SCI is marked by prominent activation of neuroinflammatory and cell death pathways, indicating a disrupted microenvironment with sustained inflammation and neuronal loss.

We further performed single‐cell RNA sequencing analysis to map intercellular signaling across major spinal cord cell types. Compared with control tissue, SCI samples showed a substantial increase in both the number and strength of cell–cell interactions, suggesting a breakdown of homeostatic regulation. Among the significantly upregulated signals, the non‐classical neurotrophic factor Midkine (MDK) stood out. Both microarray data and in vivo experiments confirmed that MDK expression sharply increases early after SCI, peaking around day 7, and then gradually declines—consistent with previous findings [21]. Moreover, Regeneration Roadmap analysis linked MDK expression with neural repair. Together, these findings suggest that MDK is an endogenous, injury‐responsive factor that participates in the early repair response after SCI. Its dynamic expression and association with regenerative signaling position MDK as a promising therapeutic target for modulating the injured spinal cord microenvironment.

### 4.2 MDK Exerts a Protective Effect Against Spinal Cord Injury by Modulating Neuroinflammation and Microglial Polarization

The primary novel finding of this study is that MDK promotes microglial polarization toward the anti‐inflammatory M2 phenotype, significantly suppressing neuroinflammation—such as by reducing TNF‐α and IL‐1β levels—and remodeling the spinal cord microenvironment to support neural repair. In this study, we demonstrated through BBB scoring and gait analysis that MDK overexpression significantly promotes motor function recovery after SCI. Furthermore, motor evoked potential (MEP) recordings and histopathological analysis confirmed that MDK facilitates structural repair of the injured spinal cord. These findings align with previous reports demonstrating that administration of recombinant human MDK improves functional recovery after SCI [12], and extend them by providing new mechanistic insight into MDK‐mediated neuroprotection. One of the key pathological features of SCI is sustained neuroinflammation, which contributes to secondary neuronal damage through the release of pro‐inflammatory cytokines such as TNF‐α and IL‐1β [22]. Our results showed that MDK overexpression significantly inhibits the pro‐inflammatory M1 microglial polarization by reducing the M1‐associated marker iNOS and suppressing the expression of these pro‐inflammatory mediators. This effect was further validated in vitro. This finding aligns with Takada et al.'s seminal work demonstrating MDK's therapeutic potential in traumatic brain injury recovery [23].

Additionally, we innovatively demonstrate that MDK‐induced M2 microglia secrete a triad of reparative factors (IL‐10, TGF‐β, and BDNF), establishing a molecular niche essential for axonal regeneration through multi‐target synergy. Microglia, as the resident immune cells of the central nervous system, critically orchestrate the inflammatory milieu following SCI. Depending on the activation state, microglia may exacerbate injury via the proinflammatory M1 phenotype or promote tissue repair through the anti‐inflammatory M2 phenotype [5]. In this study, we show that MDK overexpression robustly promotes M2 microglial polarization, as evidenced by upregulation of the M2 marker CD206 and a concurrent reduction in LPS‐induced proinflammatory cytokines IL‐1β, TNF‐α, and IL‐6. Strikingly, we further demonstrate that MDK‐induced M2 microglia secrete a coordinated triad of reparative factors—IL‐10, TGF‐β, and BDNF—collectively creating a favorable molecular niche for axonal regeneration through synergistic immunomodulatory, anti‐apoptotic, and neurotrophic effects. This integrated cytokine‐neurotrophin response represents a previously unrecognized mechanism by which MDK shapes a permissive environment for neural repair.

In parallel, MDK was found to activate critical intracellular pro‐survival pathways. Both in vivo and in vitro experiments revealed that MDK overexpression significantly upregulated phosphorylated AKT and mTOR, along with enhanced BDNF expression. These molecules are well‐established mediators of neuronal survival, synaptic plasticity, and axonal outgrowth. Notably, previous studies have implicated MDK in activating AKT signaling under hypoxic conditions to protect embryonic stem cells [24].

In summary, our results suggest that MDK not only reprograms microglial phenotype toward a reparative M2 state but also induces a cooperative release of IL‐10, TGF‐β, and BDNF to establish a multi‐targeted molecular framework that fosters axonal regeneration and functional recovery. These findings advance our understanding of the MDK–microglia axis and provide compelling rationale for targeting MDK as a therapeutic strategy in spinal cord injury.

### 4.3 Midkine Promotes Functional Recovery After Spinal Cord Injury by Enhancing Microglial Efferocytosis via LRP‐1

The third novel finding of this study is the discovery that MDK promotes LRP‐1–mediated microglial efferocytosis, thereby creating a permissive environment for neural repair following SCI. One critical factor contributing to the unfavorable neural repair environment after spinal cord injury (SCI) is the excessive accumulation of apoptotic cells within the spinal cord. These apoptotic cells and their resulting cellular debris can act as immunogenic stimuli, driving the polarization of microglia toward the proinflammatory M1 phenotype and exacerbating the neuroinflammatory response [8]. Therefore, the timely clearance of apoptotic cells in the spinal cord may represent a key mechanism for improving the spinal microenvironment. However, the role of enhanced microglial efferocytosis in promoting functional recovery after SCI remains incompletely understood.

Although both M1 and M2 microglia express phagocytic receptors, the M2 phenotype exhibits significantly enhanced efferocytic activity in clearing apoptotic cells [8, 25]. Our findings reveal that MDK overexpression significantly enhances the co‐localization of microglial marker Iba‐1 with apoptotic cell indicator cleaved‐caspase 3, concurrent with upregulation of Rac1, a key regulator of actin cytoskeleton remodeling essential for efferocytosis [26, 27], suggesting that MDK may enhance microglial clearance of apoptotic neurons. In vitro, MDK treatment restored efferocytic function in LPS‐impaired BV2 cells, reinforcing its role in promoting microglial efferocytosis. Additionally, MDK significantly increased the expression of phosphorylated AKT, mTOR, and BDNF both in vivo and in vitro, implicating this signaling axis in MDK‐mediated neuroprotection.

Efferocytosis is a receptor‐dependent process in microglia, with LRP‐1 being one of the key receptors involved [18]. Notably, LRP‐1 expression is higher in M2‐polarized microglia compared to the M1 phenotype [28], and previous studies have identified LRP‐1 as a primary receptor for MDK [10]. Our previous study found that modulating LRP‐1 function can enhance the phagocytosis of myelin debris by microglia at the site of spinal cord injury [9]. We also found that LRP‐1 is highly expressed in microglia. Based on this, we hypothesized that MDK may enhance microglial efferocytosis via LRP‐1. In support of this, our in vivo and in vitro experiments showed that MDK treatment upregulated LRP‐1 and Rac1. Furthermore, pharmacological inhibition of LRP‐1 with receptor‐associated protein (RAP) abrogated the MDK‐induced restoration of efferocytosis in BV2 cells and reversed the upregulation of Rac1. These results strongly suggest that the MDK/LRP‐1/Rac1 axis is required for the efferocytosis‐enhancing effect of MDK.

In addition to impairing phagocytosis, we also found that RAP treatment reversed the anti‐inflammatory effects of MDK, leading to increased expression of pro‐inflammatory cytokines such as IL‐1β, TNF‐α, and IL‐6. Previous research has shown that deletion of LRP‐1 in microglia activates the TLR4/NF‐κB/MAPKs and JNK signaling pathways [29, 30], thereby exacerbating microglia‐mediated neuroinflammation. Similarly, in LRP‐1^−^/^−^ atherosclerotic mouse models, efferocytosis is impaired, accompanied by increased inflammation and cell death [31]. In addition, LRP‐1 can interact with the intracellular adaptor protein Shc1 to activate the PI3K/Akt pathway, thereby promoting M2 polarization of microglia and alleviating neuroinflammation and injury following subarachnoid hemorrhage [28]. Therefore, MDK overexpression is likely to improve the spinal cord microenvironment by inducing M2 polarization and efferocytosis of microglia via LRP‐1.

These findings also imply that LRP‐1 is not only essential for MDK‐induced efferocytosis but also mediates its immunomodulatory effects. Consistently, inhibition of LRP‐1 suppressed the activation of downstream AKT/mTOR/BDNF signaling, further supporting a critical role for LRP‐1 in MDK‐mediated neuroprotection. Notably, our findings represent the first report that MDK upregulates BDNF expression through an LRP‐1–dependent mechanism, both in vivo and in vitro. Given the well‐established role of BDNF in neuronal survival, plasticity, and axonal regeneration, this novel MDK–LRP‐1–BDNF axis adds an important dimension to the neurotrophic effects of MDK and highlights a previously unrecognized molecular pathway relevant to SCI. These findings provide a strong rationale for further exploration of MDK as a promising candidate for therapeutic intervention in SCI.

### 4.4 Significance of the Study

This study systematically uncovers a novel regulatory axis—MDK/LRP‐1/efferocytosis—that plays a central role in modulating the microglial response following spinal cord injury (SCI). By integrating transcriptomic profiling, single‐cell signaling analysis, and functional validation, we demonstrate that MDK overexpression promotes microglial efferocytosis, suppresses neuroinflammation, and enhances neuroprotection. Notably, this is the first study to report that MDK can suppress neuroinflammation and upregulate BDNF expression via the LRP‐1 signaling pathway, thereby linking efferocytosis with neurotrophic support and the immune microenvironment. Compared to conventional microglia‐targeting strategies, such as minocycline or IL‐4, MDK exerts pleiotropic effects on immune resolution, neuroprotection, and functional restoration through a receptor‐specific mechanism. These findings provide a conceptual and mechanistic basis for developing MDK‐based therapies aimed at modulating the injury microenvironment and promoting recovery after SCI.

### 4.5 Limitations and Future Directions

In this study, to ensure stable MDK expression, we administered LV‐MDK 3 days prior to SCI. However, because SCI is unpredictable in clinical practice, future studies should evaluate the therapeutic efficacy of MDK when administered after injury onset, to better model clinical scenarios. Additionally, although we demonstrated that MDK promotes microglial efferocytosis and neuroprotection via LRP‐1, the exact mechanism of MDK–LRP‐1 interaction remains unclear. Our previous mechanistic studies provide crucial insights: (1) In spinal cord injury models, impaired microglial phagocytic function directly correlates with pathological cleavage of LRP‐1 ectodomain [9]; (2) Complementary research in skin wound healing demonstrates that MDK preserves LRP‐1 membrane integrity by suppressing ADAM17‐mediated proteolysis via PDI‐dependent pathways, resulting in 2.3‐fold enhanced macrophage efferocytic capacity [32]. These parallel findings suggest a conserved regulatory mechanism whereby MDK maintains LRP‐1 functionality across different myeloid cell populations and injury contexts. Third, it is also important to note that LRP‐1 is widely expressed in multiple cell types, and global inhibition of LRP‐1 may lead to adverse effects. Indeed, complete knockout of LRP‐1 leads to embryonic lethality [33]. Therefore, in the current study, we only validated MDK/LRP‐1 in vitro only. Further studies are needed to determine whether selectively inhibiting LRP‐1 expression in microglia would abolish the pro‐efferocytic effects of MDK. In addition, we observed that during the acute phase following SCI, rats overexpressing MDK exhibited significant behavioral improvement, although the MDK protein level, while showing an upward trend compared with the natural injury group, did not reach statistical significance. This discrepancy may be attributed to the rapid utilization or uptake of MDK during the acute phase of SCI. Previous studies have suggested that MDK can be internalized through endocytosis or receptor‐mediated uptake, such as LRP‐1 dependent internalization, allowing it to enter the nucleus and regulate the growth and differentiation of target cells [34, 35]. Moreover, the limited sample size (*n* = 3 per group) may have reduced the statistical power to detect subtle differences in protein levels at this early stage. Lastly, this study was conducted in rodents, and whether the findings translate to the human spinal cord remains to be explored.

In conclusion, this study reveals that the non‐classical neurotrophic factor Midkine (MDK) is markedly upregulated during the subacute phase following spinal cord injury (SCI). We demonstrate that MDK promotes functional recovery by enhancing microglial efferocytosis, suppressing neuroinflammation, and activating neuroprotective signaling pathways. Mechanistically, these effects are mediated through low‐density lipoprotein receptor‐related protein 1 (LRP‐1), which serves as a critical receptor for MDK in driving efferocytosis. Importantly, this study is the first to establish that MDK upregulates BDNF expression via the LRP‐1 pathway, linking efferocytosis to neurotrophic support. Collectively, our findings uncover a novel MDK/LRP‐1/efferocytosis signaling axis that modulates the injured spinal cord microenvironment and facilitates neurorepair. This work provides a conceptual and mechanistic foundation for the development of MDK‐based therapeutic strategies to enhance functional restoration after SCI.

## Author Contributions

Yu Wang, Zun Wang, Wen‐Tao Liu, Tong Wang, and Qi Wu designed this study; Yu Wang, Lu Fang, Chenyuan Zhai, and Jili Cai performed experiments and contributed to data analysis; Xiangzhe Li, Lijuan Zong, Chenchen Zhu, Yao Geng, Cheng Sun, Manyu Dong, and Yilun Qian conducted partial experiments; Yu Wang and Qi Wu drafted the manuscript; Yao Geng, Yan Liu, and Ying Huang completed the English language editing; Zun Wang, Tong Wang, Wen‐Tao Liu, and Qi Wu completed the review and editing. All of the authors reviewed and approved the final version of the manuscript.

## Funding

This work was supported by grants from the National Natural Science Foundation of China (82302877, 82172541), Hunan Provincial Natural Science Foundation (2023JJ30549), and Clinical Research 4310 Program of First Affiliated Hospital of the University of South China (Grant number: 20214310NHYCG07).

## Ethics Statement

All procedures were strictly performed in accordance with the regulations of the ethics committee of the International Association for the Study of Pain and the Guide for the Care and Use of Laboratory Animals (The Ministry of Science and Technology of China, 2006). All animal experiments were approved by the Nanjing Medical University Animal Care and Use Committee and were designed to minimize suffering and the number of animals used. Ethical Code for Animal Experiments: 2407080.

## Conflicts of Interest

The authors declare no conflicts of interest.

## Data Availability

The raw data of the following datasets are available from the NCBI Gene Expression Omnibus database (GEO; https://www.ncbi.nlm.nih.gov/geo/) under accession numbers GSE93561, GSE213240, and GSE172167. The datasets supporting the conclusions of this article are included in the main text and the Additional file. Additional datasets generated and/or analyzed during the current study are available from the corresponding author on reasonable request.

## References

[1] C. S. Ahuja , S. Nori , L. Tetreault , et al., “Traumatic Spinal Cord Injury‐Repair and Regeneration,” Neurosurgery 80, no. 3s (2017): S9–S22, 10.1093/neuros/nyw080. 28350947

[2] X. Zha , “Challenges and Opportunities for Repairing the Injured Spinal Cord: Inflammation, Regeneration, and Functional Reconstruction,” Regenerative Medicine Reports 2, no. 1 (2025): 36–44, 10.4103/regenmed.Regenmed-d-24-00027.

[3] A. P. Tran , P. M. Warren , and J. Silver , “The Biology of Regeneration Failure and Success After Spinal Cord Injury,” Physiological Reviews 98, no. 2 (2018): 881–917, 10.1152/physrev.00017.2017. 29513146 PMC5966716

[4] E. Boada‐Romero , J. Martinez , B. L. Heckmann , and D. R. Green , “The Clearance of Dead Cells by Efferocytosis,” Nature Reviews. Molecular Cell Biology 21, no. 7 (2020): 398–414, 10.1038/s41580-020-0232-1. 32251387 PMC7392086

[5] J. Herz , A. J. Filiano , A. T. Wiltbank , N. Yogev , and J. Kipnis , “Myeloid Cells in the Central Nervous System,” Immunity 46, no. 6 (2017): 943–956, 10.1016/j.immuni.2017.06.007. 28636961 PMC5657250

[6] Q. Wu , X. Xu , C. Zhai , et al., “Trans‐Spinal Magnetic Stimulation Attenuates Neuropathic Pain Caused by Spinal Cord Injury,” Neural Regeneration Research (2025), 10.4103/nrr.Nrr-d-24-00912. PMC13378957 40313090

[7] H. Zhang , L. Xiang , H. Yuan , and H. Yu , “PTPRO Inhibition Ameliorates Spinal Cord Injury Through Shifting Microglial M1/M2 Polarization via the NF‐κB/STAT6 Signaling Pathway,” Biochimica et Biophysica Acta—Molecular Basis of Disease 1870, no. 5 (2024): 167141, 10.1016/j.bbadis.2024.167141. 38565385

[8] S. David and A. Kroner , “Repertoire of Microglial and Macrophage Responses After Spinal Cord Injury,” Nature Reviews. Neuroscience 12, no. 7 (2011): 388–399, 10.1038/nrn3053. 21673720

[9] C. Zhai , Z. Wang , J. Cai , et al., “Repeated Trans‐Spinal Magnetic Stimulation Promotes Microglial Phagocytosis of Myelin Debris After Spinal Cord Injury Through LRP‐1,” Experimental Neurology 379 (2024): 114844, 10.1016/j.expneurol.2024.114844. 38830500

[10] Z. Z. Zhang , G. Wang , S. H. Yin , and X. H. Yu , “Midkine: A Multifaceted Driver of Atherosclerosis,” Clinica Chimica Acta 521 (2021): 251–257, 10.1016/j.cca.2021.07.024. 34331952

[11] T. Muramatsu , “Structure and Function of Midkine as the Basis of Its Pharmacological Effects,” British Journal of Pharmacology 171, no. 4 (2014): 814–826, 10.1111/bph.12353. 23992440 PMC3925020

[12] A. Muramoto , S. Imagama , T. Natori , et al., “Midkine Overcomes Neurite Outgrowth Inhibition of Chondroitin Sulfate Proteoglycan Without Glial Activation and Promotes Functional Recovery After Spinal Cord Injury,” Neuroscience Letters 550 (2013): 150–155, 10.1016/j.neulet.2013.06.025. 23811026

[13] D. M. Basso , M. S. Beattie , and J. C. Bresnahan , “Graded Histological and Locomotor Outcomes After Spinal Cord Contusion Using the NYU Weight‐Drop Device Versus Transection,” Experimental Neurology 139, no. 2 (1996): 244–256, 10.1006/exnr.1996.0098. 8654527

[14] R. Verma , J. K. Virdi , N. Singh , and A. S. Jaggi , “Animals Models of Spinal Cord Contusion Injury,” Korean Journal of Pain 32, no. 1 (2019): 12–21, 10.3344/kjp.2019.32.1.12. 30671199 PMC6333579

[15] S. Ito , M. Kawasaki , and T. Kawauchi , “Primary Culture of Dissociated Neurons From the Embryonic Cerebral Cortex,” Methods in Molecular Biology 2794 (2024): 169–175, 10.1007/978-1-0716-3810-1_14. 38630228

[16] W. Kang , T. Jin , T. Zhang , et al., “Regeneration Roadmap: Database Resources for Regenerative Biology,” Nucleic Acids Research 50, no. D1 (2022): D1085–d1090, 10.1093/nar/gkab870. 34591960 PMC8728239

[17] Z. Szondy , E. Garabuczi , G. Joós , G. J. Tsay , and Z. Sarang , “Impaired Clearance of Apoptotic Cells in Chronic Inflammatory Diseases: Therapeutic Implications,” Frontiers in Immunology 5 (2014): 354, 10.3389/fimmu.2014.00354. 25136342 PMC4117929

[18] J. Van Broeckhoven , D. Sommer , D. Dooley , S. Hendrix , and A. Franssen , “Macrophage Phagocytosis After Spinal Cord Injury: When Friends Become Foes,” Brain 144, no. 10 (2021): 2933–2945, 10.1093/brain/awab250. 34244729

[19] J. Cai , Y. Wang , C. Zhai , et al., “Body Weight‐Supported Treadmill Training Reduces Glial Scar Overgrowth in SCI Rats by Decreasing the Reactivity of Astrocytes During the Subacute Phase,” BMC Neuroscience 26, no. 1 (2025): 30, 10.1186/s12868-025-00947-7. 40295901 PMC12039159

[20] R. Gonzalez , J. Glaser , M. T. Liu , T. E. Lane , and H. S. Keirstead , “Reducing Inflammation Decreases Secondary Degeneration and Functional Deficit After Spinal Cord Injury,” Experimental Neurology 184, no. 1 (2003): 456–463, 10.1016/s0014-4886(03)00257-7. 14637115

[21] X. Qiu , Y. Guo , M. F. Liu , et al., “Single‐Cell RNA‐Sequencing Analysis Reveals Enhanced Non‐Canonical Neurotrophic Factor Signaling in the Subacute Phase of Traumatic Brain Injury,” CNS Neuroscience & Therapeutics 29, no. 11 (2023): 3446–3459, 10.1111/cns.14278. 37269057 PMC10580338

[22] I. Pineau and S. Lacroix , “Proinflammatory Cytokine Synthesis in the Injured Mouse Spinal Cord: Multiphasic Expression Pattern and Identification of the Cell Types Involved,” Journal of Comparative Neurology 500, no. 2 (2007): 267–285, 10.1002/cne.21149. 17111361

[23] S. Takada , H. Sakakima , T. Matsuyama , et al., “Disruption of Midkine Gene Reduces Traumatic Brain Injury Through the Modulation of Neuroinflammation,” Journal of Neuroinflammation 17, no. 1 (2020): 40, 10.1186/s12974-020-1709-8. 31996236 PMC6990546

[24] S. H. Lee , H. N. Suh , Y. J. Lee , B. N. Seo , J. W. Ha , and H. J. Han , “Midkine Prevented Hypoxic Injury of Mouse Embryonic Stem Cells Through Activation of Akt and HIF‐1α via Low‐Density Lipoprotein Receptor‐Related Protein‐1,” Journal of Cellular Physiology 227, no. 4 (2012): 1731–1739, 10.1002/jcp.22897. 21688265

[25] J. D. Cherry , J. A. Olschowka , and M. K. O'Banion , “Neuroinflammation and M2 Microglia: The Good, the Bad, and the Inflamed,” Journal of Neuroinflammation 11 (2014): 98, 10.1186/1742-2094-11-98. 24889886 PMC4060849

[26] Z. Ma , K. S. Thomas , D. J. Webb , et al., “Regulation of Rac1 Activation by the Low Density Lipoprotein Receptor‐Related Protein,” Journal of Cell Biology 159, no. 6 (2002): 1061–1070, 10.1083/jcb.200207070. 12499359 PMC2173989

[27] J. Tang , Y. Jin , F. Jia , et al., “Gas6 Promotes Microglia Efferocytosis and Suppresses Inflammation Through Activating Axl/Rac1 Signaling in Subarachnoid Hemorrhage Mice,” Translational Stroke Research 14, no. 6 (2023): 955–969, 10.1007/s12975-022-01099-0. 36324028

[28] J. Peng , J. Pang , L. Huang , et al., “LRP1 Activation Attenuates White Matter Injury by Modulating Microglial Polarization Through Shc1/PI3K/Akt Pathway After Subarachnoid Hemorrhage in Rats,” Redox Biology 21 (2019): 101121, 10.1016/j.redox.2019.101121. 30703614 PMC6351270

[29] Y. He , J. B. Ruganzu , H. Jin , et al., “LRP1 Knockdown Aggravates Aβ(1‐42)‐Stimulated Microglial and Astrocytic Neuroinflammatory Responses by Modulating TLR4/NF‐κB/MAPKs Signaling Pathways,” Experimental Cell Research 394, no. 2 (2020): 112166, 10.1016/j.yexcr.2020.112166. 32645395

[30] L. Yang , C. C. Liu , H. Zheng , et al., “LRP1 Modulates the Microglial Immune Response via Regulation of JNK and NF‐κB Signaling Pathways,” Journal of Neuroinflammation 13, no. 1 (2016): 304, 10.1186/s12974-016-0772-7. 27931217 PMC5146875

[31] P. G. Yancey , J. Blakemore , L. Ding , et al., “Macrophage LRP‐1 Controls Plaque Cellularity by Regulating Efferocytosis and Akt Activation,” Arteriosclerosis, Thrombosis, and Vascular Biology 30, no. 4 (2010): 787–795, 10.1161/atvbaha.109.202051. 20150557 PMC2845445

[32] L. Zong , C. Liu , L. Zhang , et al., “Remote Neuromuscular Electrical Stimulation Upregulates MDK to Enhance Macrophage Efferocytosis via LRP1 in Wound Healing,” Journal of Biomedical Research 39 (2025): 1–14, 10.7555/jbr.38.20240375. PMC13044401 40441854

[33] C. Nakajima , P. Haffner , S. M. Goerke , et al., “The Lipoprotein Receptor LRP1 Modulates Sphingosine‐1‐Phosphate Signaling and Is Essential for Vascular Development,” Development 141, no. 23 (2014): 4513–4525, 10.1242/dev.109124. 25377550 PMC4302929

[34] H. Muramatsu , K. Zou , N. Sakaguchi , S. Ikematsu , S. Sakuma , and T. Muramatsu , “LDL Receptor‐Related Protein as a Component of the Midkine Receptor,” Biochemical and Biophysical Research Communications 270, no. 3 (2000): 936–941, 10.1006/bbrc.2000.2549. 10772929

[35] Y. Shibata , T. Muramatsu , M. Hirai , et al., “Nuclear Targeting by the Growth Factor Midkine,” Molecular and Cellular Biology 22, no. 19 (2002): 6788–6796, 10.1128/mcb.22.19.6788-6796.2002. 12215536 PMC134045

